# Retinal and Choroidal Metrics Are Dynamic Markers of the Maternal Vascular Response to Pregnancy

**DOI:** 10.1161/HYPERTENSIONAHA.126.26893

**Published:** 2026-06-16

**Authors:** Kathryn Hunt, Georgia R. Morgan, Carlos Sanchez Soriano, Jamie Burke, Ylenia Giarratano, Charlene Hamid, Rosie Keane, Roseanna Jenks, Shona Low, Sarah Donaldson, Marisa Magennis, Baljean Dhillon, Rosemary C. Townsend, Tom MacGillivray, Miguel O. Bernabeu, Rebecca M. Reynolds

**Affiliations:** Institute for Neuroscience and Cardiovascular Research (K.H., G.R.M., C.S.S., C.H., R.K., S.L., S.D., M.M., B.D., T.M., R.M.R.), College of Medicine and Veterinary Medicine, University of Edinburgh, United Kingdom.; Institute for Regeneration and Repair (J.B., R.J., R.C.T., R.M.R.), College of Medicine and Veterinary Medicine, University of Edinburgh, United Kingdom.; Usher Institute (Y.G., R.C.T., M.O.B.), College of Medicine and Veterinary Medicine, University of Edinburgh, United Kingdom.

**Keywords:** choroid, placenta growth factor, pre-eclampsia, pregnancy, retina

## Abstract

**BACKGROUND:**

There is an unmet need for biomarkers that can dynamically track maternal vascular health and guide interventions in disordered pregnancies. The retina and choroid provide a window into the systemic vasculature. We aimed to characterize longitudinal trajectories of retinal and choroidal features during healthy pregnancy and explore differences associated with preeclampsia.

**METHODS:**

Overall, 251 pregnant women underwent multimodal retinal imaging with color fundus photography, scanning laser ophthalmoscopy, and optical coherence tomography at multiple antenatal (12±3 or 20±3 weeks’ gestation and 36±3 weeks’ gestation, n=183) or a single third-trimester (36±3 weeks’ gestation, n=68) time point. Retinal and choroidal vascular features were extracted using an automated pipeline. We examined gestational trajectories of these features and their associations with blood pressure change and serum angiogenic factors. Trajectories were compared for women with preeclampsia and those without placental dysfunction.

**RESULTS:**

Significant reductions in measurements of retinal vessel caliber and density and of choroidal thickness, and increases in retinal thickness measurements, were seen over healthy pregnancy. These changes were not correlated with maternal blood pressure change. Third-trimester retinal arteriolar caliber and density were weakly correlated with placental growth factor (*r*=0.32, *P*<0.001 and *r*=0.31, *P*<0.001) and soluble fms-like tyrosine kinase 1 (*r*=−0.21, *P*=0.006 and *r*=−0.33, *P*<0.001) levels. Preeclampsia was associated with significantly greater reductions in retinal arteriolar caliber (*P*=0.022 for right eye, *P*=0.019 for left) and density (*P*<0.001 for each eye) across gestation.

**CONCLUSIONS:**

Retinal and choroidal features change throughout pregnancy, and these trajectories are altered in preeclampsia.

**REGISTRATION:**

URL: https://doi.org/10.1186/ISRCTN40843826; Unique identifier: ISRCTN40843826.

**Novelty and Relevance**

**What Is New?**

This study found that measurements of choroidal thickness and retinal blood vessel thickness and density decreased, whereas measurements of retinal thickness increased, in retinal images taken across gestation from women with uncomplicated pregnancies.

Exaggerated reductions in retinal arteriolar caliber and density measurements occurred in preeclampsia.

We found associations between retinal arteriolar features in the third trimester and bloodstream levels of angiogenic factors placental growth factor and soluble fms-like tyrosine kinase 1.

**What Is Relevant?**

Blood vessels of the retina and choroid can be imaged noninvasively; monitoring how they change over time may provide insights into maternal vascular health.

**Clinical/Pathophysiological Implications?**

Retinal vessel features may be useful for risk stratification and prediction of pregnancy complications, including preeclampsia.

Associations between retinal vessel properties and serum angiogenic factors suggest potential mechanistic links between the retinal vasculature and pregnancy outcomes.

Healthy pregnancy is associated with a profound maternal cardiovascular transformation. Dramatic increases in cardiac output and circulating volume, combined with uterine vascular remodeling, facilitate a >3-fold increase in uteroplacental blood flow throughout gestation.^1^ This physiological adaptation is disordered in preeclampsia and fetal growth restriction: leading causes of obstetric mortality, which are associated with elevated lifelong cardiometabolic risk for surviving mothers and offspring.^2–5^

Incorporating vascular metrics from uterine or ophthalmic artery Doppler, or serum levels of angiogenic proteins placental growth factor (PlGF) and soluble fms-like tyrosine kinase 1 (sFlt-1), into predictive models for preeclampsia and fetal growth restriction can improve risk stratification at early and late gestations.^6–8^ However, the pathophysiological mechanisms reflected by these markers are poorly understood, and it remains challenging to anticipate disease course and time to deterioration for high-risk individuals. Indeed, a large randomized controlled trial demonstrated no clinical benefit of repeated PlGF measurement in women with suspected preeclampsia.^9^ Moreover, measurement of ultrasound Doppler indices or angiogenic protein levels requires appropriately trained sonographers and laboratory capacity, respectively. Access to these resources is limited for populations with poor or inequitable antenatal care provision (who bear the greatest burden of obstetric morbidity and mortality).^10^

There is therefore an urgent need for novel, scalable, and reliable biomarkers that can sensitively and specifically track cardiovascular adaptation to pregnancy over time and allow dynamic risk prediction for adverse outcomes.

The retinal and deeper-lying choroidal microvasculature can be directly, noninvasively visualized through the transparent structures of the eye, yielding a uniquely accessible barometer of vascular health. Characteristic changes in these vessels occur in systemic diseases including hypertension and diabetes, and applying deep learning to retinal fundus photographs can allow prediction of cardiovascular risk factors, including age, sex, smoking status, and body mass index.^11^ Vascular features at the back of the eye change dynamically and rapidly after physiological events, for example in the weeks after kidney transplantation, and are associated with risk of future cardiovascular mortality and neurodegenerative disease.^12–14^

In particular, the choroidal circulation receives ≈80% of ocular blood flow and is influenced by blood pressure, sympathetic nervous system activation, and circulating progesterone levels.^15–17^ The choroidal microvasculature may therefore provide insights into the dramatic hemodynamic, autonomic, and hormonal adaptations associated with pregnancy. In addition, excessive activation of the renin-angiotensin-aldosterone and endothelin systems has been associated with the pathophysiology of both retinopathy and preeclampsia.^18–21^ Retinal vascular changes might therefore have the potential to identify maladaptive vascular responses linked to pregnancy disorders.

In this work, we aimed to characterize normal trajectories of retinal and choroidal features during healthy pregnancy and explore differences associated with pregnancy complications. We hypothesized that altered patterns of vascular adaptation within the retina and choroid indicate the subclinical microvascular dysfunction that accompanies, or may even precede, overt maternal cardiovascular dysregulation in conditions such as preeclampsia and fetal growth restriction,^22^ and therefore that retinal and choroidal features are potential novel biomarkers of pregnancy health.

## Methods

### Data Availability

The data that support the findings of this study are available from the corresponding author on reasonable request.

### Study Design

Between April 2023 and November 2024, we enrolled pregnant women with nonanomalous singleton pregnancies into a prospective study with longitudinal and cross-sectional arms. Participants were recruited through social media advertising and from routine and high-risk antenatal clinics at the Royal Infirmary of Edinburgh, generating a cohort enriched for women with risk factors for placental dysfunction. Women with active retinal disease were excluded. Studies were performed at the University of Edinburgh according to the principles of the Declaration of Helsinki, with written informed consent from each participant. They were approved by the Brighton and Sussex research ethics committee and sponsored by the University of Edinburgh and NHS Lothian Academic and Clinical Central Office for Research and Development. The I-TEST: Novel Biomarkers in Pregnancy for Early Prediction of Stillbirth study is registered on the ISRCTN registry.

Participants in the longitudinal arms underwent retinal imaging at 2 time points antenatally. The first was at either 12 weeks’ gestation (±3 weeks) or 20 weeks’ gestation (±3 weeks). Twelve weeks was chosen as it is the usual gestation of the dating ultrasound scan in the United Kingdom, whereas 20 weeks reflects when many women in low and middle-income settings first present for antenatal care. The second retinal imaging time point was at 36 weeks’ gestation (±3 weeks), to align with the routine third-trimester contact for birth planning.

Participants in the cross-sectional arm underwent retinal imaging at 36 weeks’ gestation (±3 weeks) only.

### Study Procedures

All participants underwent examinations of both eyes in a quiet, temperature-controlled room. The right eye was imaged first for each modality. Color fundus photography (CFP) was carried out with the Canon CR-DGi nonmydriatic fundus camera, which uses a flash of white light to obtain images of the posterior pole showing the optic nerve, macula, surrounding retinal surface, and retinal blood vessels. Scanning laser ophthalmoscopy (SLO), to capture higher-contrast monochromatic images of the same structures using an infrared laser light source, and fovea-centered optical coherence tomography (OCT), generating cross-sectional images of the retina and choroid, were then performed with the Heidelberg SPECTRALIS platform (Heidelberg Engineering) as previously described.^14^ We corrected for refractive error and axial length with the OCULUS Myopia Master.

CFP images underwent automated quality control via a previously developed algorithm, QuickQual, using a rejection threshold of 0.2 to exclude images with abnormally high or low contrast (as such contrast deviations can degrade vessel segmentation performance and feature measurement accuracy).^23^ Images were then analyzed through AutoMorphalyzer,^24^ a deep learning pipeline based on AutoMorph which enables automated image processing, segmentation, and feature measurement.^25^ We extracted measurements of caliber and branching complexity for retinal arterioles and venules (Figure S1).

Measurements of retinal vessel caliber and branching pattern complexity were extracted from SLO images, and measurements of choroidal and retinal thickness were extracted from OCT images using OCTolyzer, a previously developed automated deep learning pipeline for retinal layer and vessel segmentation and feature extraction (Figures S2 and S3).^26^ Within this pipeline, choroid segmentation is carried out using the Choroidalyzer algorithm and is from the retinal pigment epithelium layer and Bruch’s membrane complex to the sclera.^27^ Measurement of retinal thickness included retinal layers between the internal limiting membrane anteriorly and the posterior boundary of the retinal pigment epithelium. SLO and OCT images were manually checked, with low-quality or poorly segmented images excluded from analysis.

A venous blood sample was taken at the third-trimester study time point for all participants in the cross-sectional study arm, and a proportion of those in the longitudinal arm, and stored at −80 °C. PlGF and sFlt-1 levels were measured using the Cobas Elecsys system (Roche Diagnostics).

Participant demographic details, blood pressure measurements recorded at routine clinical appointments, and pregnancy outcomes were extracted manually from the electronic health record and managed using REDCap electronic data capture tools hosted at the University of Edinburgh.^28^ Diagnoses of gestational hypertension and preeclampsia were made by the study team according to International Society for the Study of Hypertension in Pregnancy criteria, based on information from the health record.^29^ Fetal growth restriction was defined as per the Delphi consensus criteria.^30^

### Statistical Analysis

To demonstrate how retinal vascular features vary over the course of healthy pregnancy, we identified participants from the longitudinal study arms with no evidence of placental dysfunction. We defined this outcome as a birth after 37 completed weeks’ gestation of an infant with birthweight greater than or equal to the tenth centile (adjusted for gestational age), and an absence of preeclampsia, pregnancy-induced hypertension, fetal growth restriction, placental abruption, or stillbirth. Analysis of repeated measures was performed with a linear mixed-effects model. The fixed effect component was gestational age, with participant as the random effect modeled via a random intercept component. Regression analysis was performed for right and left eye vascular features separately. Retinal vascular features were standardized by *Z* score normalization before modeling to better compare the coefficients.

To investigate whether changes in retinal vascular features are explained by changes in maternal blood pressure over gestation, we compared changes in retinal vascular features with the change in mean arterial pressure as measured during a routine clinical appointment (selected as the closest clinical appointment to the retinal imaging visit, with the participant being excluded from this analysis if these dates were over 2 weeks apart). Linear associations are expressed as Pearson correlation coefficients.

To determine whether retinal vascular metrics are associated with serum PlGF and sFlt-1 levels, we considered participants from longitudinal and cross-sectional cohorts who gave a third-trimester venous blood sample. PlGF and sFlt-1 levels were normalized by logarithmic transformation, owing to the skewed distribution of their raw values. We express linear associations as Pearson correlation coefficients.

To explore whether the degree of retinal vascular change across gestation is associated with pregnancy outcome, we compared trajectories of retinal vascular metrics for women without placental dysfunction and those who developed preeclampsia. We carried out analysis of repeated measures using linear mixed-effects models, with gestational age, pregnancy outcome (preeclampsia or no placental dysfunction), and the interaction between gestational age and pregnancy outcome as fixed components and participant as the random effect, modeled via random intercept. Regression analysis was performed for right and left eye vascular features separately. To leverage the full data set including features from both eyes, we fitted a further linear mixed-effects model with fixed effects for gestational age, pregnancy outcome, and the interaction between the 2, and random intercepts specified for eye nested within participant.

To assess for possible confounding effects of diabetes status or aspirin use on retinal vascular changes across gestation, we carried out further linear mixed-effects modeling in the same cohort (participants with preeclampsia and those with no placental dysfunction, with right and left eye features modeled separately). Gestational age, diabetes status (defined as the presence of preexisting or gestational diabetes), and the interaction between the 2 were fixed components in our model exploring the effect of diabetes status. In the model assessing the effect of aspirin use, fixed effects were gestational age, whether regular prophylactic aspirin use was started before 16 weeks’ gestation, and the interaction between the 2. Participants were the random effect, modeled via random intercept in each analysis.

*P*<0.05 was considered statistically significant.

All analyses were conducted in R statistical software version 4.5.1.^31^

## Results

**Figure 1.**
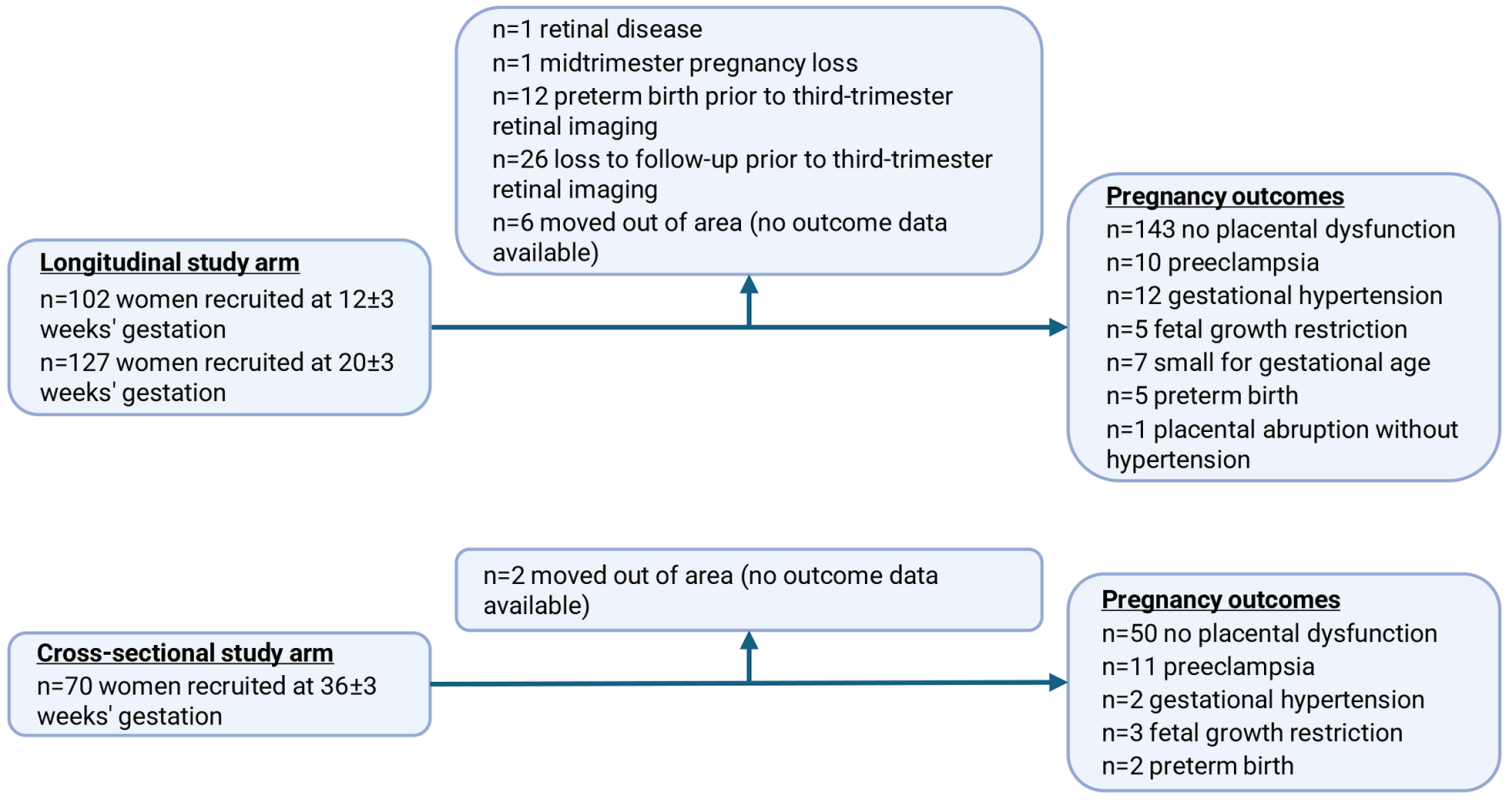
**Participant flow and pregnancy outcomes.** Small for gestational age defined as birthweight less than the tenth centile as per the International Fetal and Newborn Growth Consortium for the 21st Century standards.^32^ Preterm birth defined as birth before 37 completed weeks’ gestation.

Figure 1 shows participant flow and clinical outcomes in longitudinal and cross-sectional study arms.

### Retinal Vessel and Choroid Characteristics Evolve Dynamically Over Pregnancy

Participants without placental dysfunction recruited to the longitudinal arm at 12 weeks’ gestation (±3 weeks) and 20 weeks’ gestation (±3 weeks) were well-matched in their baseline characteristics and pregnancy outcomes and in the number of retinal images excluded due to poor image quality or segmentation (Table 1).

**Table 1.**
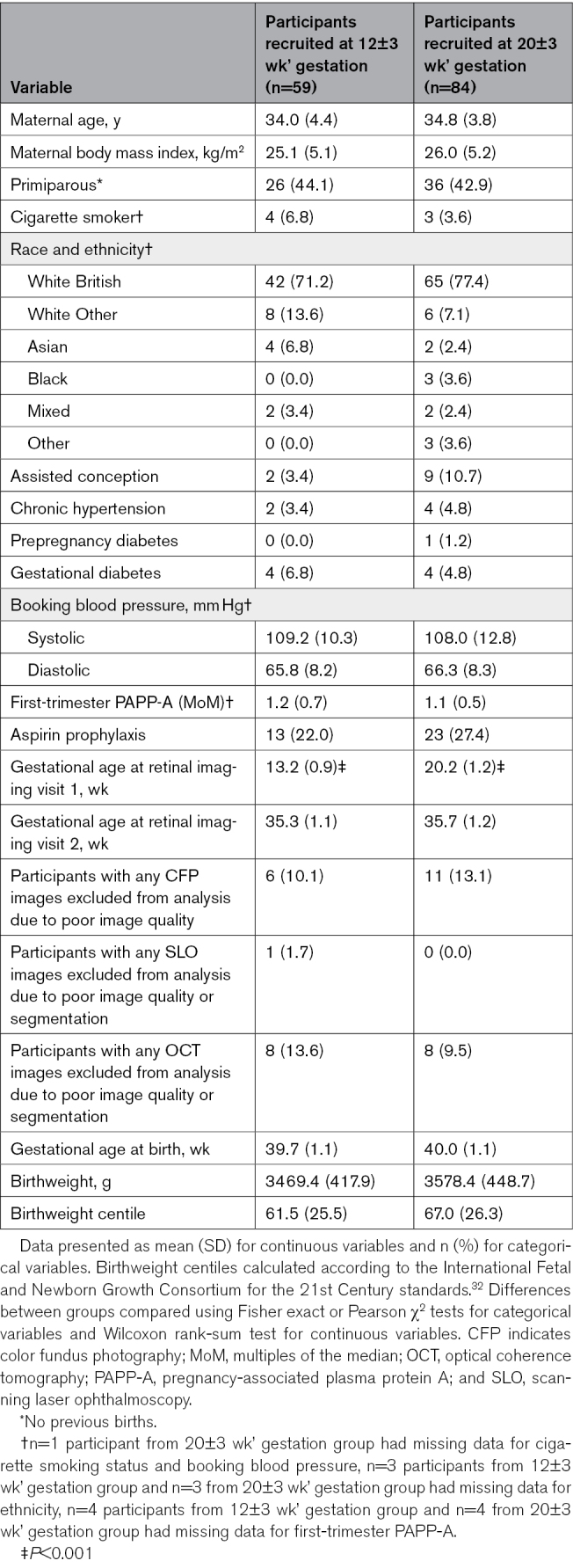
Baseline Characteristics and Pregnancy Outcomes for Longitudinal Study Arm Participants With No Placental Dysfunction

**Figure 2.**
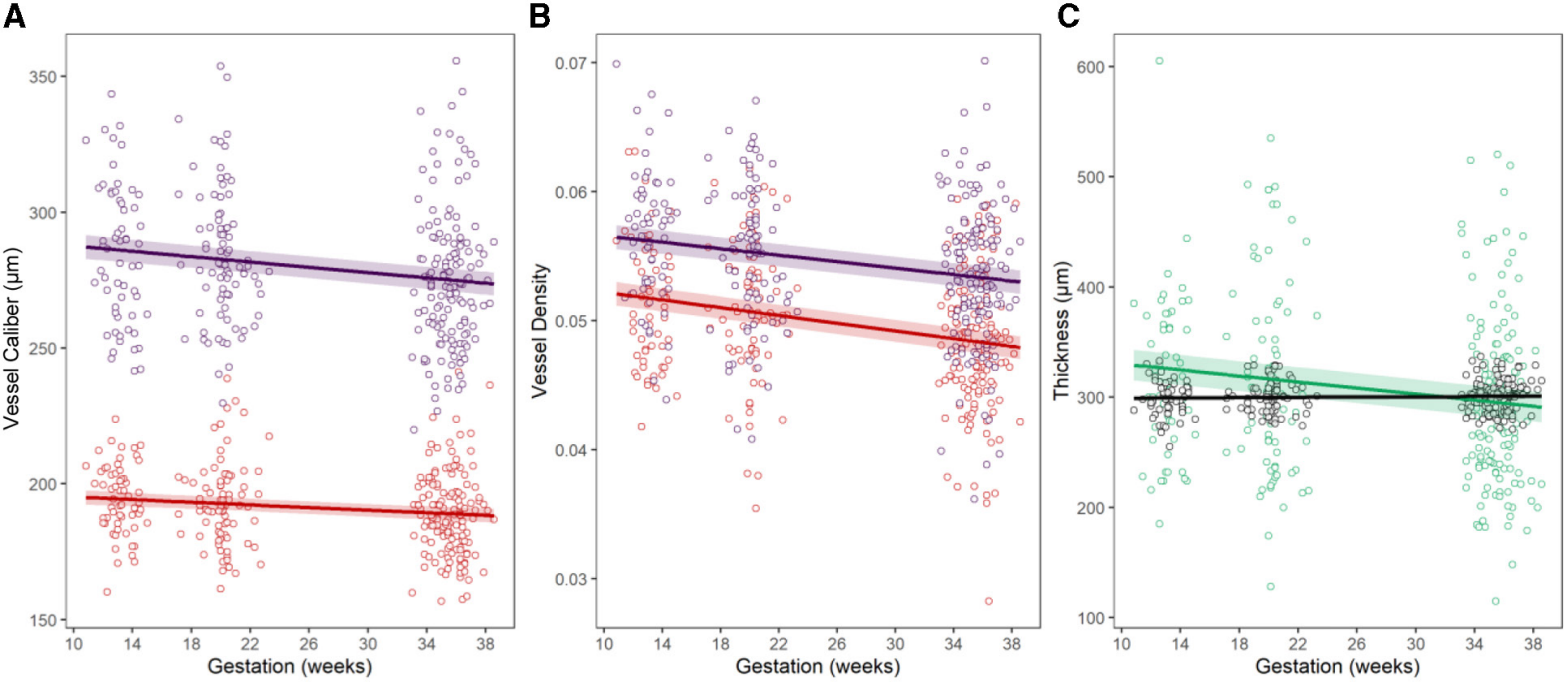
**Retinal vascular features in pregnancies without placental dysfunction.** n=143. Regression lines for linear mixed-effects models shown with 95% CIs. All features shown for right eye. **A**, Vessel caliber as measured by central retinal arteriolar equivalent and central retinal venular equivalent in scanning laser ophthalmoscopy (SLO) images, arterioles in red and venules in purple. **B**, Vessel density from SLO images, arterioles in red and venules in purple. **C**, Choroidal thickness in green and retinal thickness in black, as measured in optical coherence tomography images.

Significant reductions were seen across pregnancy in the caliber and density of retinal arterioles and venules, as measured in SLO images (Figure 2A and 2B). We also observed reductions in OCT-measured choroid thickness and increases in measurements of retinal thickness with advancing gestation (Figure 2C).

We saw similar reductions in both SLO and CFP-derived metrics of vessel caliber and density, and our findings were consistent in right and left eyes (Table S1).

### Retinal Vessel and Choroid Changes During Pregnancy Are Independent of Changes in Blood Pressure

We found minimal correlation (−0.2<*r*<0.2) between longitudinal changes in retinal vessel caliber, retinal vessel density, or choroidal or retinal thickness measurements and the change in mean arterial blood pressure, for participants without placental dysfunction (Table S2). Inclusion of participants with hypertensive disorders of pregnancy and other forms of placental dysfunction did not alter this result (Table S3).

### Third-Trimester Retinal Arteriolar Metrics Reflect Levels of Circulating Angiogenic Factors

**Figure 3.**
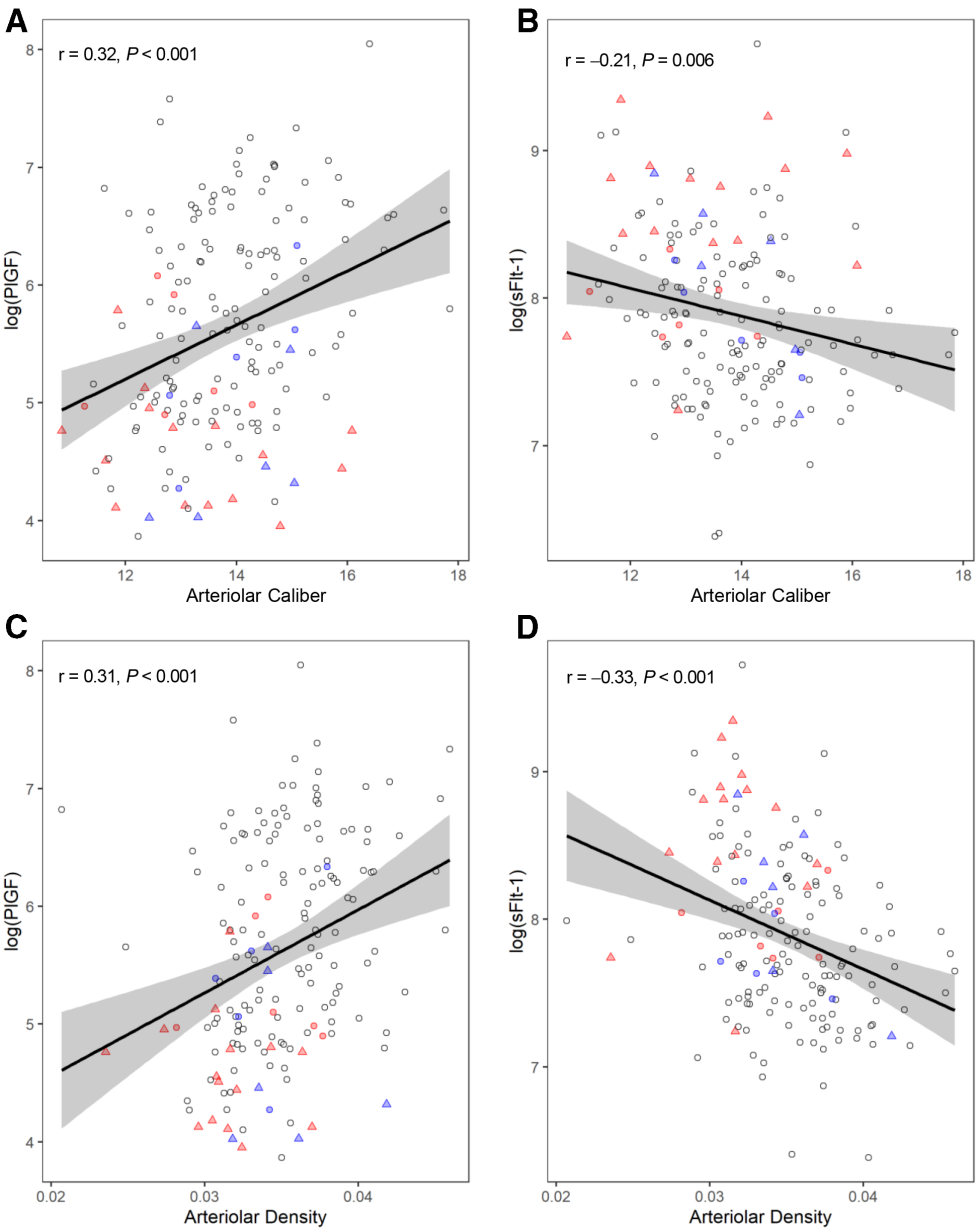
**Associations between third-trimester retinal arteriolar features and log-transformed angiogenic factor levels.** Raw placental growth factor (PlGF) and soluble fms-like tyrosine kinase 1 (sFlt-1) values in pg/mL. Uncomplicated pregnancies (n=136, white circles) shown alongside those affected by preeclampsia (n=15, red triangles), gestational hypertension (n=6, red circles), fetal growth restriction (n=6, blue triangles) and small for gestational age (n=5, blue circles). Linear regression line shown with 95% CI. All features shown for right eye. **A** and **B**, Arteriolar caliber as measured by central retinal arteriolar equivalent in color fundus photography (CFP) images. **C** and **D**, Arteriolar density from CFP images.

Baseline characteristics and clinical outcomes were similar for women who gave a blood sample for angiogenic factor measurement (n=176 across both longitudinal and cross-sectional arms), compared with the whole study cohort; although participants with gestational hypertension were underrepresented, there was no significant difference in rates of preeclampsia, fetal growth restriction, small for gestational age, placental abruption, or stillbirth (Table S4). Retinal arteriolar caliber and density measurements were weakly correlated with circulating PlGF (*r*=0.32, *P*<0.001 and *r*=0.31, *P*<0.001) and sFlt-1 levels (*r*=−0.21, *P*=0.006 and *r*=−0.33, *P*<0.001; Figure 3). These associations were consistent in right and left eyes and similar for CFP and SLO-derived vessel metrics (Table S5).

### Preeclampsia Is Associated With Exaggerated Changes in Retinal Arterioles

Women in the longitudinal cohort who developed preeclampsia had higher blood pressure at pregnancy booking, were more likely to be of Black ethnicity, and gave birth at earlier gestations to babies with lower birthweight centiles, compared with those without placental dysfunction (Table 2). There was no significant difference in their gestational ages at retinal imaging, nor the number of retinal images excluded due to poor image quality or segmentation.

**Table 2.**
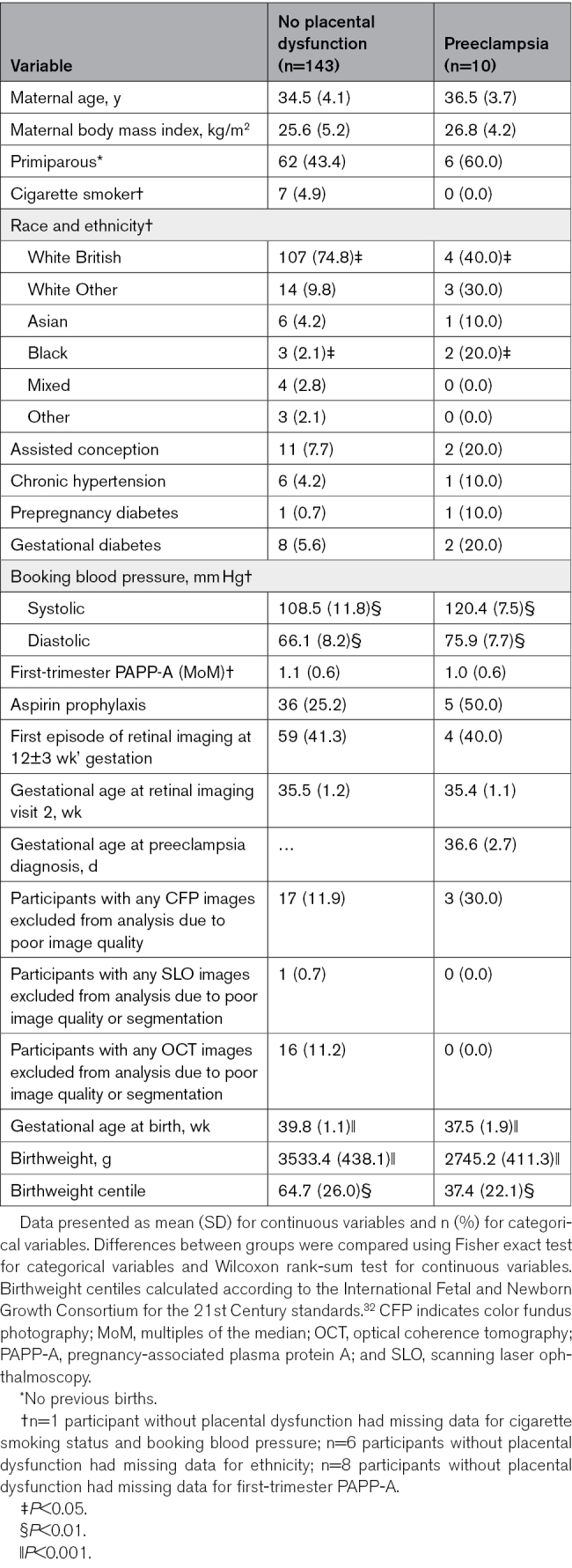
Baseline Characteristics and Pregnancy Outcomes for Participants Without Placental Dysfunction and Those With Preeclampsia

**Figure 4.**
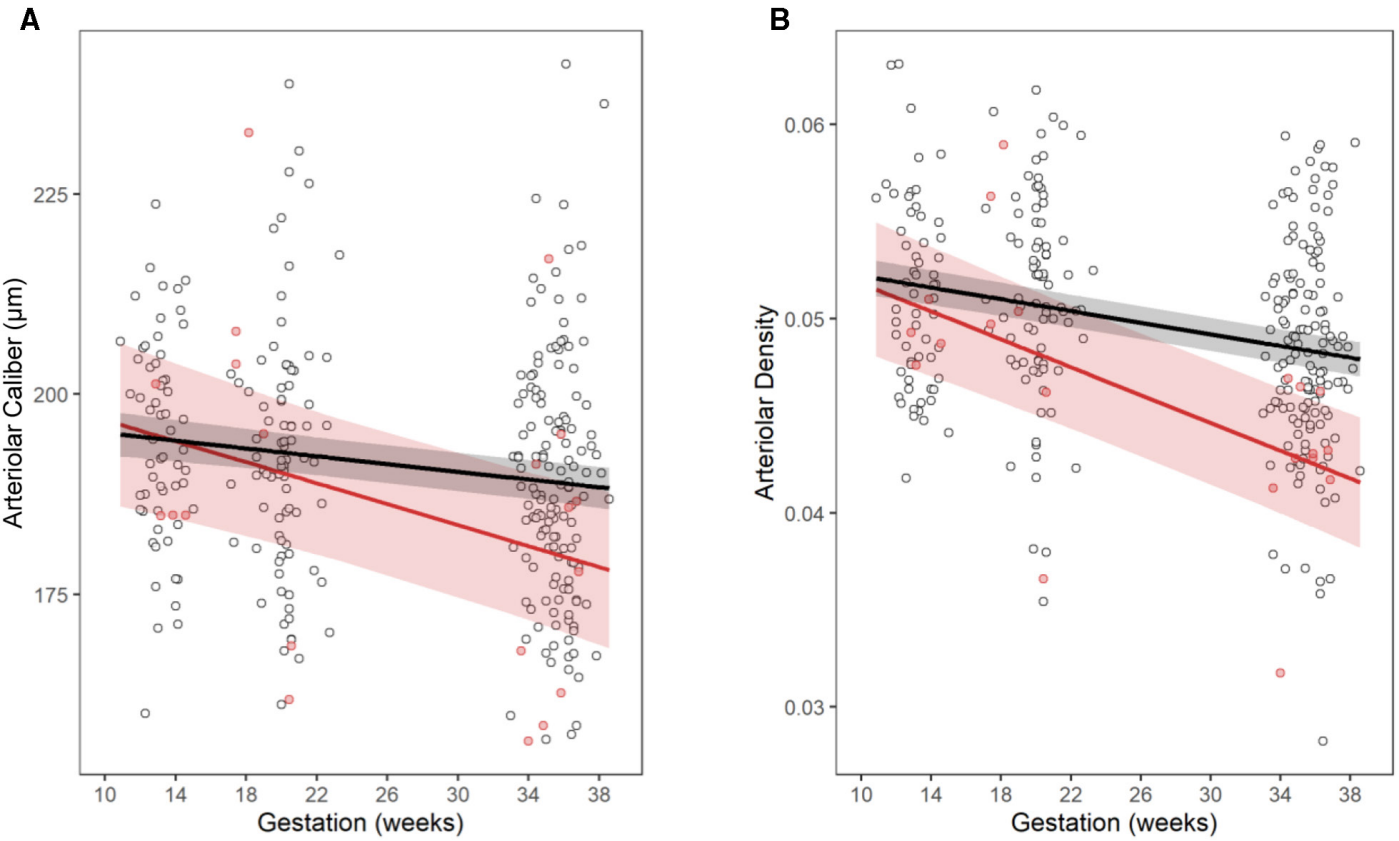
**Retinal arteriolar feature trajectories in pregnancies without placental dysfunction and those with pre-eclampsia. A**, Central retinal arteriolar equivalent and (**B**) arteriolar density, as measured in scanning laser ophthalmoscopy images, for pregnancies without placental dysfunction (n=143, black) and those with preeclampsia (n=10, red). Regression lines for linear mixed-effects models are shown with 95% CIs. All features shown for right eye. Interactions with pregnancy outcome are significant (*P*=0.022 for arteriolar caliber, *P*<0.001 for arteriolar density).

They exhibited exaggerated reductions in retinal arteriolar caliber and density across gestation, as measured in SLO images (Figure 4).

Differences in the rate of reduction in SLO-derived arteriolar caliber and density associated with preeclampsia were consistent for right and left eyes, and significantly greater reductions in retinal arteriolar density were also seen for CFP-derived metrics (Tables S6 and S7).

In linear mixed-effects models which included participant as a random effect and eye as a nested effect within participant, we also observed significant interactions with pregnancy outcome for arteriolar and venular caliber and density measurements from SLO images, for arteriolar density and caliber measurements from CFP images, and for retinal thickness as measured from OCT images (Table S8).

Within the same cohort, rates of change in image-derived retinal and choroidal features over gestation did not differ significantly by diabetes status or aspirin use (Tables S9 and S10).

## Discussion

### Principal Findings

In this study, we show that features of retinal and choroidal vessels, as measured with retinal imaging, change throughout pregnancy in a manner that is independent of gestational blood pressure change. We also demonstrate that retinal arteriolar caliber and density correlate with circulating angiogenic factor levels in the third trimester and exhibit exaggerated reductions in women with preeclampsia.

### Results in the Context of What Is Known

To our knowledge, this is the largest study to use serial multimodal retinal imaging to describe longitudinal retinal vascular changes during pregnancy, and the first to identify different trajectories in women with preeclampsia. Although previous work has identified narrower retinal vessels in women with preeclampsia, small study sample sizes, or collection of retinal imaging data at a single antenatal time point, have made it difficult to compare trends across time between healthy and complicated pregnancies.^33–35^ Likewise, a lack of longitudinal data may explain why existing studies report inconsistent findings regarding choroidal thickness in preeclamptic compared with normotensive pregnancies.^36^

Although there are few existing studies documenting longitudinal trends in retinal vascular metrics in the context of other physiological or disease states, the magnitude of change in OCT-measured choroid thickness across pregnancy in our study is comparable to those previously reported in patients after renal transplantation, and exceeds the changes observed during healthy menstrual cycles.^12,37^

### Clinical and Research Implications

Adaptation in peripheral microvascular beds occurs during pregnancy and is disordered in preeclampsia.^22^ Our finding that changes in retinal vascular features are independent of maternal blood pressure trajectories suggests that retinal vessel responses are uncoupled from pressure-related changes in larger systemic arteries. Choroidal blood flow is under autonomic nervous system control; however, it is unclear whether the reductions in OCT-measured choroidal thickness we observe throughout pregnancy reflect modulations in relative sympathetic and parasympathetic tone, or whether they are instead driven by blood volume expansion and redistribution, or tissue remodeling.^38^ Similarly, pregnancy-related increases in measured retinal thickness might be mediated by tissue growth in specific retinal layers such as the retinal nerve fiber layer, as has been suggested in previous studies, or instead may reflect intraretinal fluid accumulation.^39^ Studies to examine the behavior of specific retinal layers throughout gestation, or whether dynamic changes vary across different regions of the retina (eg, in the parafoveal and perifoveal areas), might have potential in the future to identify early markers of microvascular adaptation with greater sensitivity.

In contrast to the autonomic regulation of choroidal blood flow, retinal blood vessels autoregulate in response to local blood flow and metabolic factors. PlGF and sFlt-1 represent possible mediators of retinal vessel changes during pregnancy, given their known roles in regulating vascular smooth muscle tone and angiogenesis.^40,41^ Emerging evidence suggests that the balance of abnormalities in PlGF and sFlt-1 levels may distinguish discrete clinical subtypes of preeclampsia.^42^ We observed that PlGF was more strongly associated with retinal arteriolar caliber measurements than sFlt-1, whereas the association with arteriolar density was similar for both angiogenic factors. This difference might indicate that different disease phenotypes exhibit distinct patterns of retinal microvascular adaptation.

We propose that subclinical retinal microvascular dysfunction precedes the development of overt cardiovascular dysfunction in women with preeclampsia, as we saw exaggerated retinal arteriolar changes despite the majority (n=7, 70%) of participants with preeclampsia in the longitudinal study arm being diagnosed after their second episode of retinal imaging. Further longitudinal and mechanistic studies should explore whether differences in retinal vascular adaptation arise in parallel or in response to the aberrant placental vascularization associated with the disease.^43^ Ophthalmic artery Doppler indices have been proposed as potential prognostic markers for preeclampsia; associations between functional ophthalmic and retinal blood flow parameters and structural retinal vascular properties merit further exploration.^7^ In addition, future work to determine whether differences in retinal vascular features resolve postnatally may reveal insights into the mechanisms underlying the elevated lifetime cardiovascular risk associated with a hypertensive pregnancy.^4^

### Strengths and Limitations

We used a standardized, reproducible approach to capture and extract retinal vascular metrics from multiple imaging modalities in a large pregnant cohort at multiple gestational time points.

Women with preexisting or gestational diabetes were not excluded from the study unless retinopathy was identified. Up to 1% of pregnant women have preexisting diabetes, and up to 14% of pregnancies globally are affected by gestational diabetes.^44,45^ Inclusion of these groups and our finding that trajectories of retinal and choroidal features over gestation did not differ significantly for women with diabetes therefore increase the real-world applicability of our results.

The 5.5% incidence of preeclampsia in our longitudinal cohort is higher than background rates in the United Kingdom as estimated by epidemiological studies and likely reflects our targeted recruitment of women with known demographic and clinical risk factors.^46^ Nevertheless, our small case number may limit our ability to detect significant differences in retinal vascular trajectories for women with preeclampsia, especially given the heterogeneous nature of the condition. Although we identified significantly greater reductions in retinal arteriolar caliber and density in women with preeclampsia using SLO images, with CFP this distinction was seen only for arteriolar density. This may be because a larger number of CFP images were excluded due to poor quality or reflect the greater sensitivity of SLO for small changes in vessel caliber.

Participants in this study were predominantly of White ethnicity. Stark ethnic disparities exist in the occurrence, presentation, and outcomes of preeclampsia^47^; further studies in geographically and ethnically diverse cohorts are therefore needed to validate our findings.

Although we demonstrate the direction of change in retinal vascular features across pregnancy, longitudinal study participants underwent retinal imaging at only 2 gestational time points. This limits our ability to conclude the shape of these trajectories. Previous studies have reported nonlinear changes in retinal vessel caliber between early and late pregnancy.^48^ Future research in larger cohorts and with retinal imaging at multiple time points will help further delineate these trends.

Aspirin prophylaxis is recommended for pregnant women deemed at high risk of developing preeclampsia, based on demographic risk factors or results of first-trimester screening. Treatment with aspirin has been shown to reduce the risk of developing preterm preeclampsia and has recognized effects on endothelial function.^49^ Although this study was not powered to adjust for the effects of aspirin treatment, our finding that aspirin use did not significantly alter the trajectories of retinal and choroidal features over gestation suggests that this is unlikely to be an important confounding factor in our cohort.

Our results may have been influenced by diurnal variation in retinal vascular properties, as we did not confine retinal imaging to the same time of day for all participants and time points. However, previous work found no significant difference in choroidal thickness when measured by OCT by the same operator 3 to 4 hours apart.^12^ Studies of retinal vessel density measured by OCT angiography in healthy subjects have indicated that changes in vessel density throughout the day are within measurement variability.^50^

Our finding that changes in retinal vessel features do not correlate with changes in blood pressure across pregnancy may have arisen due to day-to-day blood pressure variability, as clinical blood pressure measurements were not taken at the same appointment as retinal imaging. However, healthy volunteer studies to assess the effect of intraoperator and interoperator variability on OCT-measured choroidal thickness demonstrated that metrics were comparable across individuals and operators for participants scanned on separate occasions 1 week apart.^12^ This suggests the impact of acute daily fluctuations in blood pressure on retinal vessel properties is unlikely to be significant.

### Conclusions

By demonstrating associations with clinically relevant angiogenic factors and altered trajectories in preeclamptic pregnancies, our findings highlight the potential of retinal vascular features as dynamic indicators of maternal vascular health.

### Perspectives

Future work in large, diverse pregnancy cohorts will establish the potential for retinal vascular features as prognostic biomarkers. CFP, SLO, and OCT are noninvasive, quick to perform, and increasingly available in optometry practices and other primary care providers. Recent advances integrating fundoscopy systems with existing smartphone camera technology may further increase the portability and accessibility of retinal imaging^51^; such developments might allow broad implementation of retinal imaging for pregnancy risk stratification, including in remote and rural settings. Disordered maternal vascular adaptation is observed in fetal growth restriction and stillbirth as well as in preeclampsia.^52,53^ It, therefore, seems plausible that biomarkers from retinal imaging, and their trajectories across pregnancy, could have a role in personalized risk stratification for these myriad manifestations of placental dysfunction.

## Article Information

### Acknowledgments

The authors are grateful for support from the Edinburgh Reproductive Tissue Biobank, NHS Lothian Laboratory Medicine, Edinburgh Imaging, and Borja Marin. Graphical abstract created in BioRender. Hunt, K. (2026) https://BioRender.com/o8xnm7r.

### Disclosures

None.

### Supplemental Material

Tables S1–S10

Figures S1–S3

## Supplementary Material


