# Supplementary material for: Retinal and Choroidal Metrics Are Dynamic Markers of the Maternal Vascular Response to Pregnancy
Source: Hypertension. 2026 Jun 16;83(8):e26893. doi: 10.1161/HYPERTENSIONAHA.126.26893 (PMC13367561; doi:10.1161/HYPERTENSIONAHA.126.26893)
Supplement: Supplementary file 1 [file hyp-83-e26893-s001.docx]

**Supplemental Material: Retinal and Choroidal Metrics Are Dynamic Markers of the Maternal Vascular Response to Pregnancy**

Kathryn Hunt^a^, Georgia R. Morgan^a^, Carlos Sanchez Soriano^a^, Jamie Burke^b^, Ylenia Giarratano^c^, Charlene Hamid^a^, Rosie Keane^a^, Roseanna Jenks^b^, Shona Low^a^, Sarah Donaldson^a^, Marisa Magennis^a^, Baljean Dhillon^a^, Rosemary C. Townsend^b,c^, Tom MacGillivray^a^, Miguel O. Bernabeu^c^, Rebecca M. Reynolds^a,b^

*a. Institute for Neuroscience and Cardiovascular Research, College of Medicine and Veterinary Medicine, University of Edinburgh, Edinburgh, UK*

*b. Institute for Regeneration and Repair, College of Medicine and Veterinary Medicine, University of Edinburgh, Edinburgh, UK*

*c. Usher Institute, College of Medicine and Veterinary Medicine, University of Edinburgh, Edinburgh, UK*

**Short title:** Retinal Vascular Metrics in Pregnancy

**Corresponding author:** Rebecca M. Reynolds

Queen’s Medical Research Institute

Edinburgh BioQuarter

47 Little France Crescent

Edinburgh EH16 4TJ

United Kingdom

Email: r.reynolds@ed.ac.uk

**Supplemental Tables**

| Feature | Right eye | | | Left eye | | |
| --- | --- | --- | --- | --- | --- | --- |
|  | Effect size of gestation | 95% confidence interval | *P* | Effect size of gestation | 95% confidence interval | *P* |
| Central retinal arteriolar equivalent (µm) (SLO) | -0.016 | (-0.02, -0.01) | <0.001 | -0.020 | (-0.03, -0.01) | <0.001 |
| Central retinal venular equivalent (µm) (SLO) | -0.019 | (-0.02, -0.01) | <0.001 | -0.019 | (-0.02, -0.01) | <0.001 |
| Average local caliber for arterioles (µm) (SLO) | -0.009 | (-0.01, 0.00) | 0.002 | -0.005 | (-0.01, 0.00) | 0.037 |
| Average local caliber for venules (µm) (SLO) | -0.011 | (-0.02, -0.01) | <0.001 | -0.009 | (-0.01, 0.00) | <0.001 |
| Arteriolar density (SLO) | -0.028 | (-0.03, -0.02) | <0.001 | -0.028 | (-0.03, -0.02) | <0.001 |
| Venular density (SLO) | -0.022 | (-0.03, -0.02) | <0.001 | -0.026 | (-0.03, -0.02) | <0.001 |
| Fractal dimension for arterioles (SLO) | -0.019 | (-0.02, -0.01) | <0.001 | -0.022 | (-0.03, -0.02) | <0.001 |
| Fractal dimension for venules (SLO) | -0.013 | (-0.02, -0.01) | <0.001 | -0.019 | (-0.02, -0.02) | <0.001 |
| Central retinal arteriolar equivalent (CFP) | -0.029 | (-0.03, -0.02) | <0.001 | -0.032 | (-0.04, -0.03) | <0.001 |
| Central retinal venular equivalent (CFP) | -0.024 | (-0.03, -0.02) | <0.001 | -0.026 | (-0.03, -0.02) | <0.001 |
| Average local caliber for arterioles (CFP) | -0.022 | (-0.03, -0.02) | <0.001 | -0.024 | (-0.03, -0.02) | <0.001 |
| Average local caliber for venules (CFP) | -0.011 | (-0.02, -0.01) | <0.001 | -0.017 | (-0.02, -0.01) | <0.001 |
| Arteriolar density (CFP) | -0.031 | (-0.04, -0.03) | <0.001 | -0.033 | (-0.04, -0.03) | <0.001 |
| Venular density (CFP) | -0.025 | (-0.03, -0.02) | <0.001 | -0.028 | (-0.03, -0.02) | <0.001 |
| Fractal dimension for arterioles (CFP) | -0.023 | (-0.03, -0.02) | <0.001 | -0.025 | (-0.03, -0.02) | <0.001 |
| Fractal dimension for venules (CFP) | -0.021 | (-0.03, -0.02) | <0.001 | -0.024 | (-0.03, -0.02) | <0.001 |
| Choroidal thickness (µm) | -0.017 | (-0.02, -0.01) | <0.001 | -0.016 | (-0.02, -0.01) | <0.001 |
| Retinal thickness (µm) | 0.005 | (0.00, 0.01) | <0.001 | 0.005 | (0.00, 0.01) | <0.001 |

**Table S1:** Effect sizes of gestation (weeks) in linear-mixed effects regression models for scanning laser ophthalmoscopy (SLO), color fundus photography (CFP), and optical coherence tomography (OCT)-derived retinal and choroidal features in right and left eyes, in pregnancies without placental dysfunction.

| Feature | Right eye | | Left eye | |
| --- | --- | --- | --- | --- |
|  | *r* | *P* | *r* | *P* |
| Central retinal arteriolar equivalent (µm) | -0.097 | 0.39 | 0.048 | 0.67 |
| Central retinal venular equivalent (µm) | -0.117 | 0.30 | -0.083 | 0.47 |
| Arteriolar density | -0.032 | 0.78 | 0.163 | 0.14 |
| Venular density | 0.100 | 0.38 | 0.132 | 0.24 |
| Choroidal thickness (µm) | -0.018 | 0.88 | 0.044 | 0.69 |
| Retinal thickness (µm) | -0.037 | 0.75 | -0.089 | 0.43 |

**Table S2:** Associations between change in scanning laser ophthalmoscopy (SLO) and optical coherence tomography (OCT)-measured retinal and choroidal features and change in blood pressure over gestation for pregnancies without placental dysfunction (n=82).

| Feature | Right eye | | Left eye | |
| --- | --- | --- | --- | --- |
|  | *r* | *P* | *r* | *P* |
| Central retinal arteriolar equivalent (µm) | -0.092 | 0.35 | 0.047 | 0.63 |
| Central retinal venular equivalent (µm) | -0.210 | 0.03 | -0.143 | 0.15 |
| Arteriolar density | -0.161 | 0.10 | 0.114 | 0.25 |
| Venular density | -0.040 | 0.68 | 0.022 | 0.82 |
| Choroidal thickness (µm) | 0.015 | 0.88 | 0.055 | 0.58 |
| Retinal thickness (µm) | -0.025 | 0.80 | -0.037 | 0.70 |

**Table S3:** Associations between change in scanning laser ophthalmoscopy (SLO) and optical coherence tomography (OCT)-measured retinal and choroidal features and change in blood pressure over gestation, for all participants in longitudinal study arms with available blood pressure data (n=107 with outcomes as follows: n=82 no placental dysfunction, n=7 preeclampsia, n=7 gestational hypertension, n=4 fetal growth restriction, n=5 small for gestational age, n=1 preterm birth, n=1 placental abruption).

| Variable | Participants who gave a serum sample for angiogenic factor measurement (n=176) | Entire study population (n=251) |
| --- | --- | --- |
| Maternal age (years) | 34.4 (3.9) | 34.4 (4.1) |
| Maternal body mass index (kg/m^2^) | 26.9 (6.4) | 26.7 (6.1) |
| Primiparous^†^ | 88 (50.0) | 121 (48.2) |
| Cigarette smoker^‡^ | 11 (6.3) | 12 (4.8) |
| Ethnicity^‡^  White British  White Other  Asian  Black  Mixed  Other | 131 (74.9)  17 (9.7)  6 (3.4)  4 (2.3)  5 (2.9)  5 (2.9) | 180 (72.0)  31 (12.4)  9 (3.6)  5 (2.0)  9 (3.6)  6 (2.4) |
| Assisted conception | 19 (10.8) | 28 (11.2) |
| Chronic hypertension | 8 (4.5) | 12 (4.8) |
| Prepregnancy diabetes | 3 (1.7) | 4 (1.6) |
| Gestational diabetes | 13 (7.4) | 17 (6.8) |
| Booking blood pressure (mmHg)^‡^  Systolic  Diastolic | 111.1 (12.6)  67.6 (8.7) | 110.7 (12.3)  67.6 (8.7) |
| First-trimester PAPP-A (MoM) ^‡^ | 1.1 (0.6) | 1.1 (0.6) |
| Aspirin prophylaxis | 56 (31.8) | 78 (31.1) |
| Gestational age at third-trimester retinal imaging (weeks) | 35.6 (1.4) | 35.5 (1.3) |
| Participants with any third-trimester CFP images excluded from analysis due to poor image quality | 13 (7.4) | 21 (8.4) |
| Participants with any third-trimester SLO images excluded from analysis due to poor image quality or segmentation | 1 (0.6) | 1 (0.4) |
| Participants with any third-trimester OCT images excluded from analysis due to poor image quality or segmentation | 13 (7.4) | 16 (6.4) |
| Preeclampsia | 16 (9.1) | 21 (8.4) |
| Gestational age at preeclampsia diagnosis (weeks) ^‡^ | 36.6 (2.2) | 36.5 (2.2) |
| Gestational hypertension | 6 (3.4)^\|\|^ | 14 (5.6)^\|\|^ |
| Gestational age at gestational hypertension diagnosis (days) ^‡^ | 37.1 (2.8) | 37.4 (4.0) |
| Fetal growth restriction | 7 (4.0) | 9 (3.6) |
| Small for gestational age | 6 (3.4) | 9 (3.6) |
| Placental abruption*^#^* | 0 (0.0) | 2 (2.7) |
| Stillbirth*^#^* | 1 (0.6) | 1 (0.4) |
| Gestational age at birth (weeks) | 39.5 (1.5) | 39.4 (1.5) |
| Birthweight (g) | 3437.8 (554.7) | 3415.3 (553.0) |
| Birthweight centile | 61.5 (29.9) | 60.6 (29.3) |

**Table S4:** Baseline characteristics and pregnancy outcomes for participants who gave a third-trimester serum sample for angiogenic factor measurement, alongside the entire study cohort. Data presented as mean (standard deviation) for continuous variables and n (%) for categorical variables. Birthweight centiles calculated according to the International Fetal and Newborn Growth Consortium for the 21st Century standards^32^. Differences between groups compared using Fisher’s exact or Pearson’s chi-squared tests for categorical variables and Wilcoxon rank sum test for continuous variables. CFP indicates color fundus photography; MoM, multiples of the median; OCT, optical coherence tomography; PAPP-A, pregnancy-associated plasma protein A; and SLO, scanning laser ophthalmoscopy.

^||^P<0.5 ^†^no previous births ^‡^n=1 participant who gave a third-trimester serum sample had missing data for cigarette smoking status and booking blood pressure, n=8 participants who gave a serum sample and n=3 who did not has missing data for ethnicity, n=7 participants who gave a serum sample and n=3 who did not had missing data for first-trimester PAPP-A, gestational age at preeclampsia and gestational hypertension diagnoses are the mean for participants with these diagnoses. ^#^n=1 participant with preeclampsia and n=1 participant without hypertensive disease had placental abruptions (neither participant gave third-trimester serum samples), n=1 participant experienced an antenatal stillbirth, in the context of a preeclampsia diagnosis.

| Feature | Correlation with log(PlGF (pg/mL)) | | | | Correlation with log(sFlt-1 (pg/mL)) | | | |
| --- | --- | --- | --- | --- | --- | --- | --- | --- |
|  | Right eye | | Left eye | | Right eye | | Left eye | |
|  | *r* | *P* | *r* | *P* | *r* | *P* | *r* | *P* |
| Central retinal arteriolar equivalent (CFP) | 0.32 | <0.001 | 0.30 | <0.001 | -0.21 | 0.006 | -0.21 | 0.008 |
| Central retinal venular equivalent (CFP) | 0.18 | 0.022 | -0.01 | 0.950 | -0.04 | 0.650 | 0.01 | 0.880 |
| Arteriolar density (CFP) | 0.31 | <0.001 | 0.32 | <0.001 | -0.33 | <0.001 | -0.37 | <0.001 |
| Venular density (CFP) | 0.12 | 0.110 | 0.09 | 0.250 | -0.12 | 0.120 | -0.12 | 0.120 |
| Central retinal arteriolar equivalent (µm) (SLO) | 0.27 | <0.001 | 0.21 | 0.006 | -0.15 | 0.045 | -0.07 | 0.400 |
| Central retinal venular equivalent (µm) (SLO) | 0.07 | 0.370 | 0.07 | 0.340 | 0.04 | 0.580 | 0.04 | 0.630 |
| Arteriolar density (SLO) | 0.26 | <0.001 | 0.24 | 0.002 | -0.23 | 0.002 | -0.23 | 0.003 |
| Venular density (SLO) | 0.03 | 0.730 | 0.03 | 0.720 | -0.01 | 0.870 | 0.04 | 0.570 |
| Choroidal thickness (µm) | 0.01 | 0.850 | 0.03 | 0.720 | 0.04 | 0.590 | 0.06 | 0.410 |
| Retinal thickness (µm) | 0.07 | 0.370 | 0.08 | 0.320 | -0.16 | 0.043 | -0.18 | 0.018 |

**Table S5:** Associations between change in color fundus photography (CFP), scanning laser ophthalmoscopy (SLO) and optical coherence tomography (OCT)-measured retinal and choroidal features and log-transformed angiogenic factor levels in the third trimester. PlGF indicates placental growth factor; and sFlt-1, soluble fms-like tyrosine kinase 1.

| Feature | Right eye | | | | | | | | |
| --- | --- | --- | --- | --- | --- | --- | --- | --- | --- |
|  | Gestation (weeks) | | | Preeclampsia | | | Interaction with preeclampsia | | |
|  | Effect size | 95% confidence interval | *P* | Effect size | 95% confidence interval | *P* | Effect size | 95% confidence interval | *P* |
| Central retinal arteriolar equivalent (µm) (SLO) | -0.016 | (-0.02, -0.01) | <0.001 | 0.372 | (-0.46, 1.21) | 0.385 | -0.027 | (-0.05, 0.00) | 0.022 |
| Central retinal venular equivalent (µm) (SLO) | -0.019 | (-0.02, -0.01) | <0.001 | 0.727 | (-0.05, 1.51) | 0.07 | -0.011 | (-0.03, 0.01) | 0.278 |
| Arteriolar density (SLO) | -0.027 | (-0.03, -0.02) | <0.001 | 0.301 | (-0.44, 1.04) | 0.427 | -0.037 | (-0.05, -0.02) | <0.001 |
| Venular density (SLO) | -0.022 | (-0.03, -0.02) | <0.001 | 0.204 | (-0.54, 0.95) | 0.594 | -0.013 | (-0.03, 0.00) | 0.135 |
| Central retinal arteriolar equivalent (CFP) | -0.028 | (-0.03, -0.02) | <0.001 | -0.198 | (-1.01, 0.61) | 0.634 | -0.007 | (-0.03, 0.01) | 0.489 |
| Central retinal venular equivalent (CFP) | -0.023 | (-0.03, -0.02) | <0.001 | 0.638 | (-0.15, 1.42) | 0.114 | -0.012 | (-0.03, 0.01) | 0.207 |
| Arteriolar density (CFP) | -0.031 | (-0.04, -0.03) | <0.001 | -0.146 | (-0.95, 0.65) | 0.722 | -0.023 | (-0.04, 0.00) | 0.028 |
| Venular density (CFP) | -0.025 | (-0.03, -0.02) | <0.001 | -0.043 | (-0.84, 0.76) | 0.917 | -0.007 | (-0.03, 0.01) | 0.490 |
| Choroidal thickness (µm) | -0.017 | (-0.02, -0.01) | <0.001 | 0.210 | (-0.48, 0.90) | 0.551 | 0.003 | (-0.01, 0.01) | 0.626 |
| Retinal thickness (µm) | 0.005 | (0.00, 0.01) | <0.001 | 0.008 | (-0.67, 0.68) | 0.981 | -0.008 | (-0.02, 0.00) | 0.033 |

**Table S6:** Linear mixed effects models for scanning laser ophthalmoscopy (SLO), color fundus photography (CFP), and optical coherence tomography (OCT)-derived retinal and choroidal features in the right eye. Pregnancies affected by preeclampsia were compared to those without placental dysfunction.

| Feature | Left eye | | | | | | | | |
| --- | --- | --- | --- | --- | --- | --- | --- | --- | --- |
|  | Gestation (weeks) | | | Preeclampsia | | | Interaction with preeclampsia | | |
|  | Effect size | 95% confidence interval | *P* | Effect size | 95% confidence interval | *P* | Effect size | 95% confidence interval | *P* |
| Central retinal arteriolar equivalent (µm) (SLO) | -0.019 | (-0.03, -0.01) | <0.001 | 0.762 | (-0.10, 1.62) | 0.085 | -0.030 | (-0.05, -0.01) | 0.019 |
| Central retinal venular equivalent (µm) (SLO) | -0.018 | (-0.02, -0.01) | <0.001 | 1.317 | (0.49, 2.15) | 0.002 | -0.023 | (-0.05, 0.00) | 0.057 |
| Arteriolar density (SLO) | -0.028 | (-0.03, -0.02) | <0.001 | 0.355 | (-0.38, 1.09) | 0.344 | -0.032 | (-0.05, -0.02) | <0.001 |
| Venular density (SLO) | -0.026 | (-0.03, -0.02) | <0.001 | 0.012 | (-0.74, 0.76) | 0.976 | -0.019 | (-0.04, 0.00) | 0.038 |
| Central retinal arteriolar equivalent (CFP) | -0.032 | (-0.04, -0.03) | <0.001 | 0.154 | (-0.68, 0.99) | 0.719 | -0.028 | (-0.05, -0.01) | 0.014 |
| Central retinal venular equivalent (CFP) | -0.026 | (-0.03, -0.02) | <0.001 | 0.319 | (-0.52, 1.16) | 0.456 | -0.007 | (-0.03, 0.01) | 0.497 |
| Arteriolar density (CFP) | -0.032 | (-0.04, -0.03) | <0.001 | 0.238 | (-0.59, 1.07) | 0.576 | -0.033 | (-0.06, -0.01) | 0.004 |
| Venular density (CFP) | -0.028 | (-0.03, -0.02) | <0.001 | -0.104 | (-0.97, 0.76) | 0.815 | -0.005 | (-0.03, 0.02) | 0.700 |
| Choroidal thickness (µm) | -0.015 | (-0.02, -0.01) | <0.001 | 0.444 | (-0.23, 1.12) | 0.199 | -0.003 | (-0.01, 0.01) | 0.543 |
| Retinal thickness (µm) | 0.005 | (0.00, 0.01) | <0.001 | -0.189 | (-0.87, 0.49) | 0.59 | -0.005 | (-0.02, 0.01) | 0.350 |

**Table S7:** Linear mixed effects models for scanning laser ophthalmoscopy (SLO), color fundus photography (CFP), and optical coherence tomography (OCT)-derived retinal and choroidal features in the left eye. Pregnancies affected by preeclampsia were compared to those without placental dysfunction.

| Feature | Gestation (weeks) | | | Preeclampsia | | | Interaction with preeclampsia | | |
| --- | --- | --- | --- | --- | --- | --- | --- | --- | --- |
|  | Effect size | 95% confidence interval | *P* | Effect size | 95% confidence interval | *P* | Effect size | 95% confidence interval | *P* |
| Central retinal arteriolar equivalent (µm) (SLO) | -0.017 | (-0.02, -0.01) | <0.001 | 0.574 | (-0.10, 1.25) | 0.098 | -0.029 | (-0.05, -0.01) | 0.001 |
| Central retinal venular equivalent (µm) (SLO) | -0.018 | (-0.02, -0.01) | <0.001 | 1.036 | (0.38, 1.69) | 0.002 | -0.017 | (-0.02, 0.00) | 0.023 |
| Arteriolar density (SLO) | -0.027 | (-0.03, -0.02) | <0.001 | 0.340 | (-0.29, 0.97) | 0.289 | -0.036 | (-0.05, -0.02) | <0.001 |
| Venular density (SLO) | -0.024 | (-0.03, -0.02) | <0.001 | 0.118 | (-0.51, 0.75) | 0.713 | -0.016 | (-0.03, 0.00) | 0.010 |
| Central retinal arteriolar equivalent (CFP) | -0.030 | (-0.03, -0.03) | <0.001 | -0.052 | (-0.73, 0.63) | 0.882 | -0.016 | (-0.03, 0.00) | 0.033 |
| Central retinal venular equivalent (CFP) | -0.024 | (-0.03, -0.02) | <0.001 | 0.487 | (-0.18, 1.15) | 0.153 | -0.010 | (-0.02, 0.00) | 0.161 |
| Arteriolar density (CFP) | -0.032 | (-0.04, -0.03) | <0.001 | 0.059 | (-0.63, 0.75) | 0.868 | -0.029 | (-0.04, -0.01) | <0.001 |
| Venular density (CFP) | -0.026 | (-0.03, -0.02) | <0.001 | -0.068 | (-0.77, 0.63) | 0.849 | -0.006 | (-0.02, 0.01) | 0.433 |
| Choroidal thickness (µm) | -0.016 | (-0.02, -0.01) | <0.001 | 0.324 | (-0.31, 0.96) | 0.320 | 0.000 | (-0.01, 0.01) | 0.936 |
| Retinal thickness (µm) | 0.005 | (0.00, 0.01) | <0.001 | -0.088 | (-0.73, 0.55) | 0,789 | -0.007 | (-0.01, 0.00) | 0.044 |

**Table S8:** Linear mixed effects models for scanning laser ophthalmoscopy (SLO), color fundus photography (CFP), and optical coherence tomography (OCT)-derived retinal and choroidal features, with participant as a random effect and eye as a nested effect within participant. Pregnancies affected by preeclampsia were compared to those without placental dysfunction.

| Feature | Right eye | | | | | | | | |
| --- | --- | --- | --- | --- | --- | --- | --- | --- | --- |
|  | Gestation (weeks) | | | Diabetes status | | | Interaction with diabetes status | | |
|  | Effect size | 95% confidence interval | *P* | Effect size | 95% confidence interval | *P* | Effect size | 95% confidence interval | *P* |
| Central retinal arteriolar equivalent (µm) (SLO) | -0.017 | (-0.02, -0.01) | <0.001 | 0.605 | (-0.17, 1.38) | 0.127 | -0.008 | (-0.03, 0.01) | 0.438 |
| Central retinal venular equivalent (µm) (SLO) | -0.020 | (-0.03, -0.02) | <0.001 | -0.112 | (-0.83, 0.61) | 0.761 | 0.008 | (-0.01, 0.03) | 0.351 |
| Arteriolar density (SLO) | -0.029 | (-0.03, -0.02) | <0.001 | 0.311 | (-0.39, 1.01) | 0.387 | -0.009 | (-0.03, 0.01) | 0.304 |
| Venular density (SLO) | -0.023 | (-0.03, -0.02) | <0.001 | 0.121 | (-0.81, 0.57) | 0.731 | 0.002 | (-0.01, 0.02) | 0.765 |
| Central retinal arteriolar equivalent (CFP) | -0.029 | (-0.03, -0.02) | <0.001 | -0.116 | (-0.84, 0.60) | 0.753 | 0.000 | (-0.02, 0.02) | 0.977 |
| Central retinal venular equivalent (CFP) | -0.024 | (-0.03, -0.02) | <0.001 | -0.029 | (-0.73, 0.67) | 0.934 | -0.001 | (-0.02, 0.01) | 0.904 |
| Arteriolar density (CFP) | -0.032 | (-0.04, -0.03) | <0.001 | 0.168 | (-0.55, 0.89) | 0.649 | -0.009 | (-0.03, 0.01) | 0.355 |
| Venular density (CFP) | -0.026 | (-0.03, -0.02) | <0.001 | -0.145 | (-0.85, 0.56) | 0.690 | 0.005 | (-0.01, 0.02) | 0.543 |
| Choroidal thickness (µm) | -0.017 | (-0.02, -0.01) | <0.001 | 0.042 | (-0.60, 0.68) | 0.898 | 0.004 | (-0.01, 0.01) | 0.361 |
| Retinal thickness (µm) | 0.005 | (0.00, 0.01) | <0.001 | -0.278 | (-0.90, 0.34) | 0.381 | -0.004 | (-0.01, 0.00) | 0.237 |

**Table S9:** Linear mixed effects models for scanning laser ophthalmoscopy (SLO), color fundus photography (CFP), and optical coherence tomography (OCT)-derived retinal and choroidal features in the right eye amongst participants with preeclampsia and no placental dysfunction. Women with diabetes (preexisting or gestational) were compared to those without diabetes.

| Feature | Right eye | | | | | | | | |
| --- | --- | --- | --- | --- | --- | --- | --- | --- | --- |
|  | Gestation (weeks) | | | Aspirin use | | | Interaction with aspirin use | | |
|  | Effect size | 95% confidence interval | *P* | Effect size | 95% confidence interval | *P* | Effect size | 95% confidence interval | *P* |
| Central retinal arteriolar equivalent (µm) (SLO) | -0.017 | (-0.02, -0.01) | <0.001 | 0.191 | (-0.67, 0.29) | 0.435 | -0.003 | (-0.02, 0.01) | 0.701 |
| Central retinal venular equivalent (µm) (SLO) | -0.019 | (-0.02, -0.01) | <0.001 | -0.093 | (-0.54, 0.35) | 0.683 | -0.002 | (-0.01, 0.01) | 0.753 |
| Arteriolar density (SLO) | -0.028 | (-0.03, -0.02) | <0.001 | 0.009 | (-0.42, 0.44) | 0.967 | -0.007 | (-0.02, 0.00) | 0.206 |
| Venular density (SLO) | -0.023 | (-0.03, -0.02) | <0.001 | 0.039 | (-0.38, 0.46) | 0.859 | -0.001 | (-0.01, 0.01) | 0.817 |
| Central retinal arteriolar equivalent (CFP) | -0.028 | (-0.03, -0.02) | <0.001 | -0.400 | (-0.85, 0.04) | 0.080 | -0.004 | (-0.02, 0.01) | 0.522 |
| Central retinal venular equivalent (CFP) | -0.025 | (-0.03, -0.02) | <0.001 | -0.469 | (-0.890, -0.04) | 0.035 | -0.004 | (-0.01, 0.01) | 0.428 |
| Arteriolar density (CFP) | -0.030 | (-0.04, -0.02) | <0.001 | -0.031 | (-0.48, 0.42) | 0.893 | -0.009 | (-0.02, 0.00) | 0.119 |
| Venular density (CFP) | -0.026 | (-0.03, -0.02) | <0.001 | -0.242 | (-0.69, 0.20) | 0.288 | 0.003 | (-0.01, 0.01) | 0.557 |
| Choroidal thickness (µm) | -0.018 | (-0.02, -0.02) | <0.001 | -0.137 | (-0.53, 0.26) | 0.496 | 0.005 | (0.00, 0.01) | 0.099 |
| Retinal thickness (µm) | 0.005 | (0.00, 0.01) | <0.001 | 0.246 | (-0.13, 0.62) | 0.202 | -0.001 | (0.00, 0.00) | 0.737 |

**Table S10:** Linear mixed effects models for scanning laser ophthalmoscopy (SLO), color fundus photography (CFP), and optical coherence tomography (OCT)-derived retinal and choroidal features in the right eye amongst participants with preeclampsia and no placental dysfunction. Women who took regular aspirin prophylaxis during pregnancy were compared to those who did not.

**Supplemental Figures**


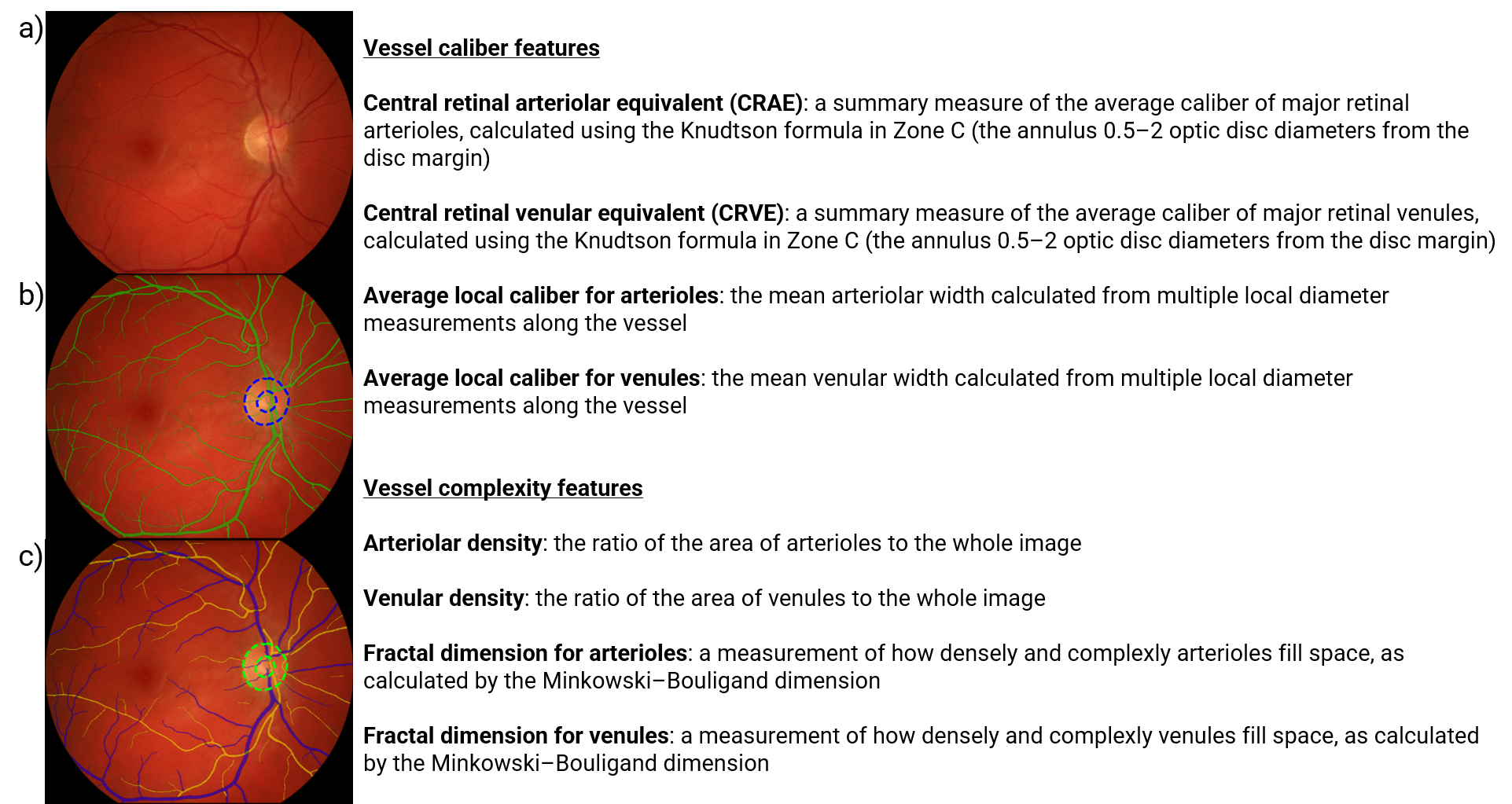


**Figure S1:** Retinal vascular features extracted from color fundus photography images. Original image shown in (a), automated vessel segmentation shown in (b), and segmentation of arterioles in yellow and venules in purple shown in (c). Vessel caliber features reported in pixel units.


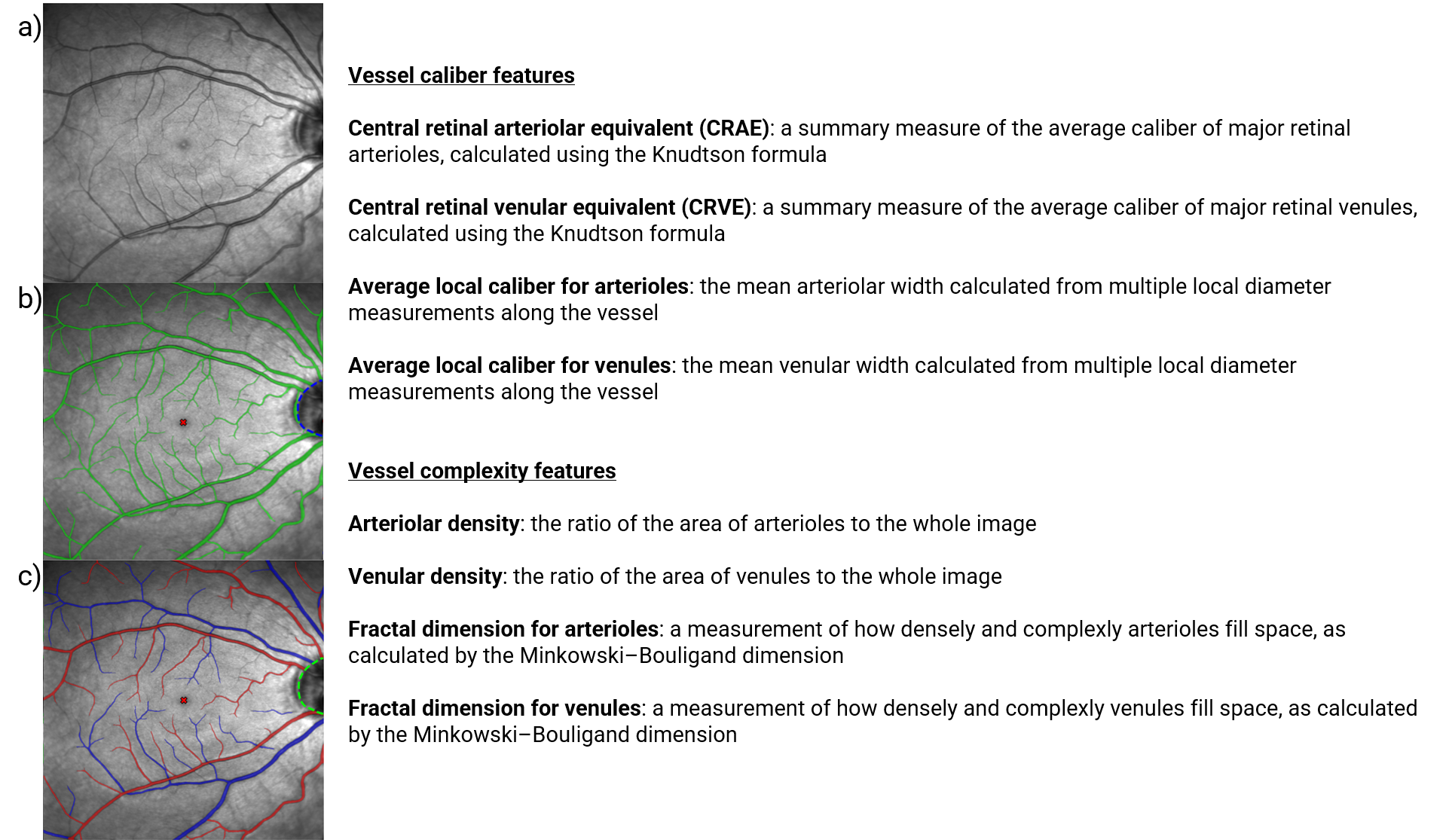


**Figure S2:** Retinal vascular features extracted from scanning laser ophthalmoscopy images. Original image shown in (a), automated vessel segmentation shown in (b), and segmentation of arterioles in red and venules in blue shown in (c). Detected foveal position shown by red cross.


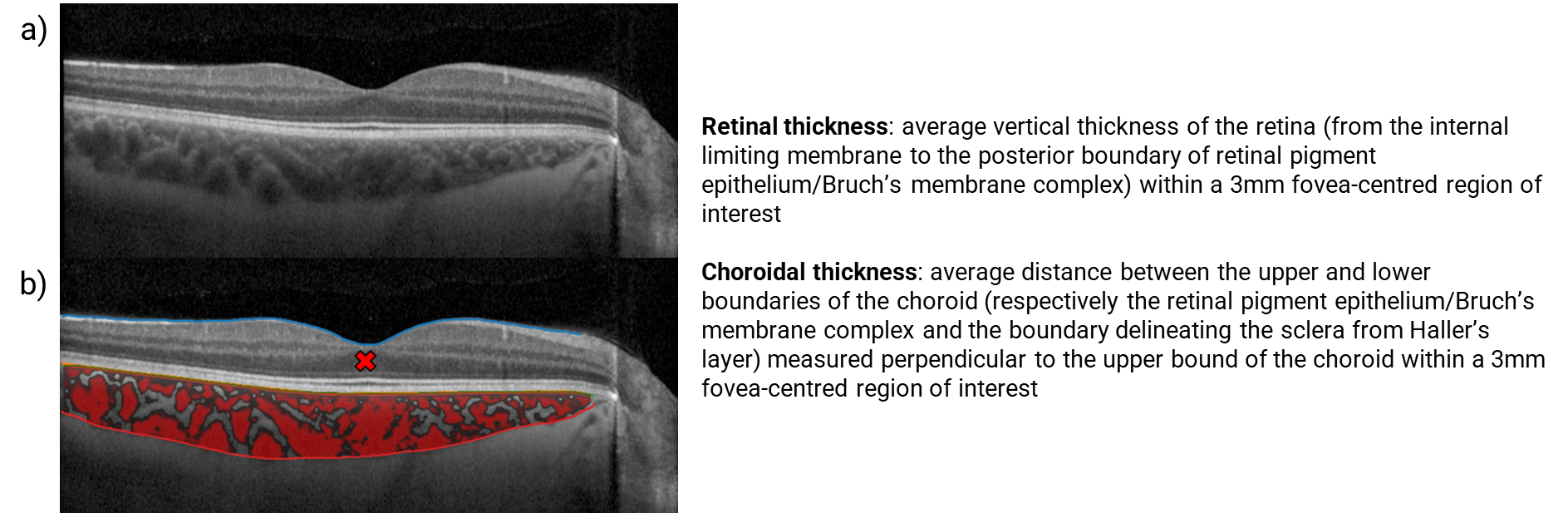


**Figure S3:** Retinal and choroidal features extracted from optical coherence tomography images. Original foveal-centred B-scan shown in (a), retinal segmentation (between upper blue line and upper red line) and choroid segmentation (between upper and lower red lines) shown in (b). Foveal position shown by red cross.
